# A New Serum Macrophage Checkpoint Biomarker for Innate Immunotherapy: Soluble Signal-Regulatory Protein Alpha (sSIRPα)

**DOI:** 10.3390/biom12070937

**Published:** 2022-07-04

**Authors:** Yoanna V. Vladimirova, Marie K. Mølmer, Kristian W. Antonsen, Niels Møller, Nikolaj Rittig, Marlene C. Nielsen, Holger J. Møller

**Affiliations:** 1 Department of Clinical Biochemistry, Aarhus University Hospital, 8200 Aarhus N, Denmark; yoavla@rm.dk (Y.V.V.); mariemoelmer@gmail.com (M.K.M.); kriant@rm.dk (K.W.A.); machne@rm.dk (M.C.N.); 2 Department of Endocrinology and Internal Medicine, Medical/Steno Research Laboratories, Aarhus University Hospital, 8200 Aarhus N, Denmark; niels.moeller@clin.au.dk (N.M.); nikolaj.rittig@clin.au.dk (N.R.); 3 Department of Clinical Medicine, Aarhus University, 8200 Aarhus N, Denmark

**Keywords:** macrophage, immunotherapy, biomarker, SIRPα, CD47, checkpoint, cancer

## Abstract

**Background and Aims:** The macrophage “don’t eat me” pathway CD47/SIRPα is a target for promising new immunotherapy. We hypothesized that a soluble variant of SIRPα is present in the blood and may function as a biomarker. **Methods:** Monocyte derived macrophages (MDMs) from human buffy-coats were stimulated into macrophage subtypes by LPS and IFN-γ (M1), IL-4 and IL-13 (M2a), IL-10 (M2c) and investigated using flow cytometry. Soluble SIRPα (sSIRPα) was measured in cell cultures and serum by Western blotting and an optimized ELISA. Serum samples were obtained from 120 healthy individuals and from 8 individuals challenged by an LPS injection. **Results:** All macrophage phenotypes expressed SIRPα by flowcytometry, and sSIRPα was present in all culture supernatants including unstimulated cells. M1 macrophages expressed the lowest level of SIRPαand released the highest level of sSIRPα (*p* < 0.05). In vivo, the serum level of sSIRPα increased significantly (*p* < 0.0001) after an LPS challenge in humans. The median concentration in healthy individuals was 28.7 µg/L (19.8–41.1, 95% reference interval), and 20.5 µg/L in an IFCC certified serum reference material. The protein was stable in serum for prolonged storage and repeated freeze/thawing. **Conclusions:** We demonstrate that sSIRPα is produced constitutively and the concentration increases upon macrophage activation both in vitro and in vivo. It is present in human serum where it may function as a biomarker for the activity of tumor-associated macrophages (TAMs), and for monitoring the effect of immunotherapy.

## 1. Introduction

T-cell based immunotherapy has revolutionized the treatment of certain cancers; however, in most cancers significant response rates are still not reached. Research is therefore focused now on ways to involve the innate immune system in cancer therapy [1]. Tumor-associated macrophages (TAMs) can phagocytize cancer cells and stimulate adaptive immunity; however, TAMs predominantly suppress antitumor immunity. This is in part due to cancer cells expressing so-called “don’t eat me” signals that inhibit phagocytosis through receptor-binding to TAMs [1].

**Figure 1:**
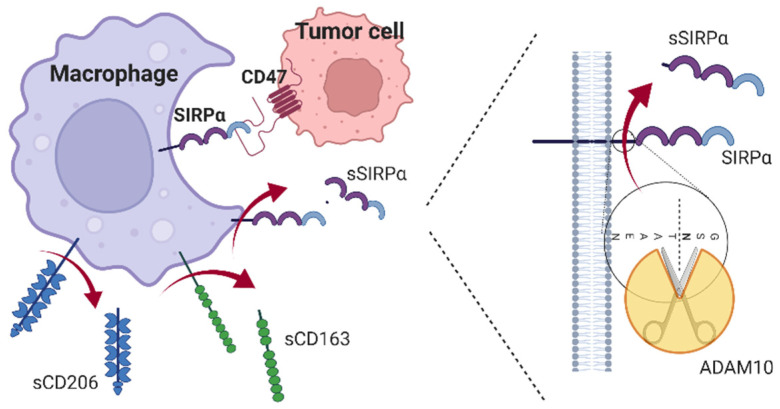
CD47 on tumor cells protects against phagocytosis. It interacts with SIRPα on the surface of TAMs resulting in decreased macrophage phagocytic capacity. Activation of macrophages leads to shedding by metalloproteases of soluble receptors (sCD163, sCD206) that function as biomarkers of macrophage activation in the blood. Soluble SIRPα (sSIRPα) is thought to be the product of extracellular domain shedding by the action of ADAM10 that, upon activation, cleaves SIRPα at a juxta-membrane position (15). Created with BioRender.com.

An important signal system is the CD47/signal regulatory protein alpha (SIRPα) pathway, which is the target for several inhibitory drugs that are currently being investigated in clinical trials including soluble variants of SIRPα to outcompete macrophage binding to CD47 on the tumor-cells [2]. CD47 is widely expressed and protects cells against phagocytosis. It is upregulated on cancer cells and interacts with SIRPα on the surface of TAMs. This leads to downstream signaling that dampens macrophage phagocytic capacity [1] (Figure 1).

SIRPα is an approximately 90 kDa glycoprotein with four possible N-linked glycosylation sites (https://www.uniprot.org/uniprot/P78324, accessed on 20 June 2022). In humans, the SIRP gene family consists of SIRPα, SIRPβ1, SIRPβ2, SIRPγ, and SIRPδ and is clustered on chromosome 20p133 [3,4,5,6]. SIRPα consists of three extracellular immunoglobulin superfamily (IgSF) domains, a single-span transmembrane region and a tyrosine-rich cytoplasmic tail. The IgSF domains including one IgV-like and two IgC-like domains are proximal to the N terminal. The IgV-like domain is responsible for binding antigens such as the IgV-like domain of CD47. The cytoplasmic tail contains four tyrosine residues, which upon phosphorylation recruit the tyrosine phosphatases SHP-1 and SHP-2 resulting in downstream signaling. SIRPα is normally expressed on neural tissue and on myelomonocytic cells, including monocytes, macrophages, granulocytes, dendritic cells and their precursors [4,7,8,9,10]. SIRPα is also expressed on TAMs, and recent evidence links high SIRPα expression to poor survival in patients with esophageal squamous cell carcinoma [11]. Thus, SIRPα is both a potential therapeutic target, and a potential biomarker in cancer.

A soluble form of SIRPα has been detected in vitro [12,13,14]. This protein is thought to be the product of extracellular domain shedding by the action of a disintegrin and metalloproteinase domain-containing protein 10 (ADAM10) that, upon activation, cleaves SIRPα at a juxta-membrane position to produce soluble SIRPα (sSIRPα) [12] (Figure 1). This soluble form may be present in human blood, and its concentration could reflect the level of intra-tumoral immune activation.

We hypothesized that sSIRPα is present as a stable protein in human blood and may be a useful biomarker with the potential for monitoring immunotherapy. Furthermore, we wanted to establish a robust method for its determination.

## 2. Methods

### 2.1. Human Monocyte-Derived Macrophages

Buffy coats (one sample, ∼50 mL) from five healthy donors were obtained from the blood bank at the Department of Clinical Immunology, Aarhus University Hospital, Aarhus, Denmark (project no. 0094). According to Danish law, the use of anonymized buffy coats does not require specific ethical approval.

Monocytes were isolated from the buffy coats as described previously [15]. In short, the buffy coats were diluted 1:2 in 0.9% NaCl and the peripheral blood mononuclear cells (PBMCs) were isolated using density gradient centrifugation on a Histopaque-1077 gradient (Sigma-Aldrich, Munich, Germany). Monocytes were subsequently isolated from the PBMCs using EasySep™ Human Monocyte Isolation Kit (Stemcell Technologies, Vancouver, Canada) according to the manufacturer’s protocol.

For monocyte-derived macrophage (MDM) differentiation, the isolated monocytes were cultured in non-treated T-75 flasks in complete maturation media (RPMI-1640 with 10% FCS, 100 U/100 µg/mL penicillin/streptomycin (all from ThermoFisher scientific, Waltham, MA, USA)), 10 ng/mL macrophage colony-stimulating factor (M-CSF), and 1 ng/mL granulocyte-macrophage colony-stimulating factor (GM-CSF) (both from Peprotech, Stockholm, Sweden) for 5 days. Media were changed every 2–3 days.

After MDM differentiation, the MDMs were stimulated with either 100 ng/mL LPS and 20 ng/mL IFN-γ (M1 stimulation), 10 ng/mL IL-4 and 10 ng/mL IL-13 (M2a stimulation), 10 ng/mL IL-10 (M2c stimulation) (all cytokines from Peprotech) or left untreated (M0) for 24 h to promote MDM polarization.

### 2.2. Flow Cytometry

After harvesting the MDM subtypes from the wells, unspecific antibody binding was blocked using human IgG (Beriglobin, CSL Behring, King of Prussia, PA, USA) at a final concentration of 100 µg/mL. The cells were stained with antibodies in a stain buffer consisting of PBS with 0.5% BSA and 0.09% NaN_3_. Each subtype was split and stained with live/dead fixable dye near IF (ThermoFisher Scientific) and anti-SIRPα FITC (clone 15-414, conc. 6 µg/mL, Biolegend, San Diego, CA, USA), to assess SIRPα expression. Alternatively, in order to validate the MDM polarization, with: live/dead dye and anti-CD45 AF700 (clone HI30, conc. 2.5 µg/mL, BD Biosciences, Erembodegem, Belgium); anti-CD11b BV510 (clone IRCF44, conc. 1.5 µg/mL, Biolegend); anti-CD80 V450 (clone L307.4, conc. 0.2 µg/mL, Biolegend); anti-CD163 PE (clone Mac2-158, conc. 0.4 µg/mL, Trillium Diagnostics, Brewer, ME, USA); anti-SIRPα FITC (clone 15-414, conc. 6 µg/mL); anti-TLR2 PE-Vio770 (REA109, conc. 2.2 µg/mL, Miltenyi Biotech, Bergish Gladbach, Germany); and anti-CD206 APC (clone 15-2, conc. 3 µg/mL, Biolegend). Cells were incubated with antibodies at 4 °C for 30 min, after which they were washed in stain buffer and fixed in PBS with 0.9% formaldehyde.

The cells were analyzed on a Navios Flow Cytometer (Beckman Coulter, Brea, CA, USA). Spectral overlap compensation was performed using single-stained antibody capture beads, BD™ CompBeads Plus (BD Biosciences), MACS^®^ Comp Bead Kit anti-REA (Miltenyi Biotech), and ArC™ Amine Reactive Compensation Bead Kit for Live/dead (ThermoFisher). Data processing was performed using FlowJo 10.7 for Windows (FlowJo, LLC, Ashland, OR, USA). The gating strategy for both panels is presented in Supplemental Figure S1.

### 2.3. Optimized ELISA

One hundred microliters of a polyclonal anti-human SIRPα antibody (1.5 mg/L, R&D Systems, catalogue number AF4546, diluted in 20 mM carbonate-bicarbonate buffer pH 9.6 mg/mL) was coated onto microtiter wells (Nunc Maxisorp) and incubated at 4 °C overnight. The wells were washed three times in PBS, and subsequently blocked with 100 µL PBS-albumin (10 mM, 0.5 M NaCl, 0.25% (*v*/*v*) Tween 20, 0.2% (*w*/*v*) bovine serum albumin Sigma A-4503), pH 7.2).

Plates were washed and subsequently 100 μL of the sample, diluted in PBS-albumin, was added and incubated for 1 h. The wells were then washed, and 100 μL of in-house biotinylated monoclonal anti-SIRPα antibody (Abcam, Cambridge, UK, EPR22930-163) was diluted to 0.4 mg/L in PBS-albumin, added, and incubated for 1 h.

After washing, 100 μL of avidin-lysozyme mixture (12 mL PBS-albumin pH 7.2 + 120 μL lysozyme (Sigma-Aldrich L6876, dilution 20 mg/m) + 6 μL avidin-POD (Sigma-Aldrich, St. Louis, MO, USA, A7419)) was added and incubated for 1 h. The wells were washed and 100 μL TMB One (KEM-EN-TEC, Taastrup, Denmark, catalogue number 4380A) was added. After incubating for 20 min, 50 μL of 1 M phosphoric acid was added, and the plates were read at 450/620 nm in a microtiter plate reader (ThermoFischer Scientific, Multiscan^TM^ FC).

A standard curve spanning a range from 0.125-8 µg/L was prepared from a recombinant human SIRPα (R&D Systems, 9378-SA-050). A human serum quality control sample (HK12, DEKS, Glostrup, Denmark)) was included in all runs, diluted 1:100. One hundred and twenty human serum samples were obtained from the blood bank at the Department of Clinical Immunology, Aarhus University Hospital, Aarhus, Denmark (project no. 304) and diluted 1:100. The concentration of sSIRPα was also determined in a certified reference material (ERM^®^- DA470k/IFCC). Recombinant human SIRPβ1 (R&D Systems, 9978-SB) and recombinant human SIRPγ (R&D Systems, 9999-SB) were used to test for specificity.

### 2.4. Western Blotting

Serum (diluted 1:50) and MDM culture supernatants were analyzed by sodium dodecyl sulphate-polyacrylamide gel electrophoresis (4–12% bis tris NuPAGETM Invitrogen Waltham, MA, USA catalogue number NPO335), according to the supplier’s instructions. Proteins were blotted onto PVDF membranes, blocked for 1 h, and incubated overnight at 4 °C with sheep anti-human SIRPα antibody (R&D Systems, catalogue number AF4546, 0.4 µg/mL for serum and 1.6 µg/mL for supernatants). The membrane was subsequently incubated for 1 h at room temperature (RT) with rabbit anti-sheep horseradish peroxidase, and developed using enhanced chemiluminescence. BioRad, Hercules, CA, USA, Precision Plus Protein™ WesternC™ Blotting Standards, catalogue no. #1610376, were used.

### 2.5. Human LPS Exposure

We measured sSIRPα in plasma samples from a previously conducted human randomized crossover trial (clinicaltrials.gov registration no. NCT01705782) investigating eight young, lean, healthy male volunteers for six consecutive hours following LPS injection [16]. A single dose of 1 ng/kg bodyweight of Escherichia coli endotoxin was given at time = 0 min (The United States Pharmacopeial Convention, Inc., Rockville, Maryland, lot H0K354;). Blood samples were collected at baseline, 180 min, and 360 min following LPS exposure. Plasma was stored at −20 °C until analysis.

The concentrations of the soluble endocytic receptors sCD163 (haptoglobin-hemoglobin receptor) and sCD206 (mannose receptor) were measured using in-house ELISA as described [17,18].

### 2.6. Statistical Methods

Multiple comparisons were performed by ANOVA followed by Tukey’s multiple comparison test. Non-parametric tests were used to investigate the effects of freeze–thaw cycles, storage temperature, storage time, and time to centrifugation. For sample stability before centrifugation and freeze–thaw stability, the Friedman test was used. The stability of pipetted samples, at different storage temperatures for up to one month, was tested with Wilcoxon matched-pairs signed rank test. A parametric 95% reference interval was established by calculating the mean ±1.96 σ from log-transformed data. Statistical analysis was performed using GraphPad Prism 8 and Analyse-it for Microsoft Excel.

## 3. Results

### 3.1. Expression of SIRPα and Shedding of sSIRPα by Macrophage Subtypes In Vitro

The expression and shedding of SIRPα was investigated in human MDMs polarized into inflammatory M1 (LPS, IFN) or anti-inflammatory M2a (IL4, IL13), and M2c (IL10) macrophage subtypes. As expected, TLR-2 and CD80 were upregulated in M1 cells, CD206 upregulated in M2a cells, and CD163 upregulated in M2c cells (Supplemental Figure S2).

**Figure 2:**
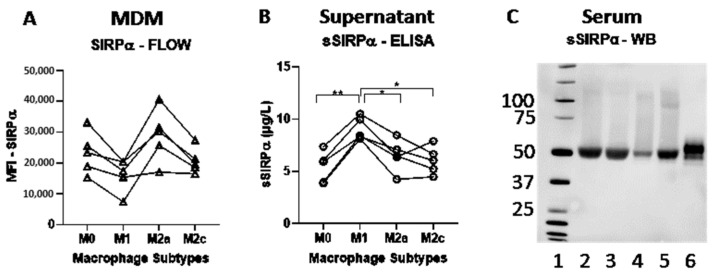
**Expression and shedding of SIRPα**. (**A**) Monocyte derived macrophages (M0) were polarized into macrophage subtypes by LPS and IFN-γ (M1), IL-4 and IL13 (M2a), IL-10 (M2c) and the expression of SIRPα was investigated using flowcytometry. (**B**) Soluble SIRPα (sSIRPα) was measured in the cell cultures by ELISA. (* *p* < 0.05, ** *p* < 0.01). (**C**) Western blotting of serum-samples from four individuals (Lane 2–5: samples, Lane 6: Recombinant extracellular SIRPα).

All MDM subtypes expressed SIRPα at levels measurable by flow cytometry (Table 1), and there were significantly different expression levels (one-way ANOVA *p* = 0.0087) between subtypes, with the lowest expression on M1 MDMs (Table 1, Figure 2a).

Soluble SIRPα was present in supernatants from all MDM subtypes, indicating a constitutive production (Figure 2b). Statistically significantly higher levels were measured in supernatants from M1 MDMs, in accordance with proteolytic shedding by metalloproteases upon LPS stimulation (Table 2, Figure 2b).

Soluble SIRPα was detectable by Western blotting in human serum showing an apparent molecular weight similar to recombinant extracellular SIRPα (Figure 2c).

### 3.2. Shedding of sSIRPα In Vivo

**Figure 3:**
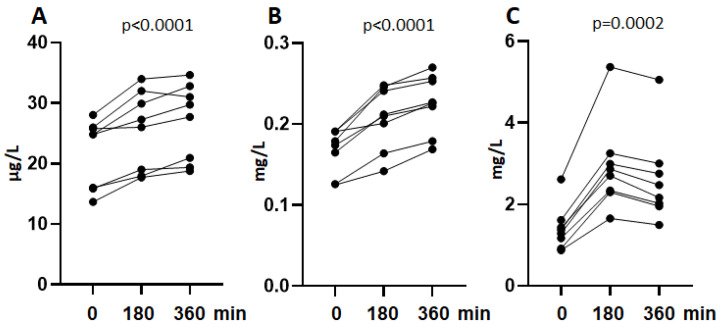
**Shedding of sSIRPα in vivo**. The concentration of sSIRPα (**A**), sCD206 (**B**), and sCD163 (**C**) were measured at t = 0, t = 180 and t = 360 min in blood samples from 8 healthy individuals. Participants were injected with a bolus of LPS at t = 0. Repeated measures ANOVA.

To investigate if LPS mediated SIRPα shedding could be demonstrated in vivo, we measured the concentration of sSIRPα in consecutive blood samples from eight healthy individuals injected with a bolus of LPS in a randomized crossover design (Figure 3a). We compared the kinetic to two endocytic receptors sCD206 (Figure 3b) and sCD163 (Figure 3c), also known to be shed from macrophages upon protease activity. The plasma level of sSIRPα increased by 16 and 20% after 3 and 6 h (repeated measures one-way ANOVA, *p* < 0.0001) which was comparable to sCD206 (21 and 23%, respectively, *p* < 0.0001), but lower than for sCD163 (109 and 41%, respectively, *p* = 0.0002). The level of sCD163 reached maximum already after 3 h, whereas sSIRPα and sCD206 showed a weaker and more prolonged response.

### 3.3. Validation of an ELISA for sSIRPα

We explored five commercially available antibodies against sSIRPα and established and optimized an ELISA using a polyclonal coating-antibody and a monoclonal biotinylated secondary antibody. Within run imprecision was 3.3% CV (n = 16, 1 run). Intermediary (between run) imprecision was 5.9% CV (n = 34, 17 runs). The LOD was below 0.015 μg/L. The assay was linear in serum dilutions ranging from 1:10 to 1:2430, R^2^ = 0.99, covering a concentration range of 0.015–3.7 µg/L. Mean recovery of recombinant SIRPα added to patient samples was 92% (n = 10, range 80–99). Neither rhSIRPβ nor rhSIRPγ (84.5% sequence similarity and 77.6% sequence similarity to SIRPα in the extracellular region, respectively) added to serum samples reacted in the assay (<0% recovery at tested concentrations). We determined the concentration of sSIRPα in a certified reference material from IFCC (ERM^®^-DA470k/IFCC) to 20.5 µg/L +/− 1.4 µg/L (2SD, n = 9, 3 runs).

### 3.4. Preanalytical Factors

**Figure 4:**
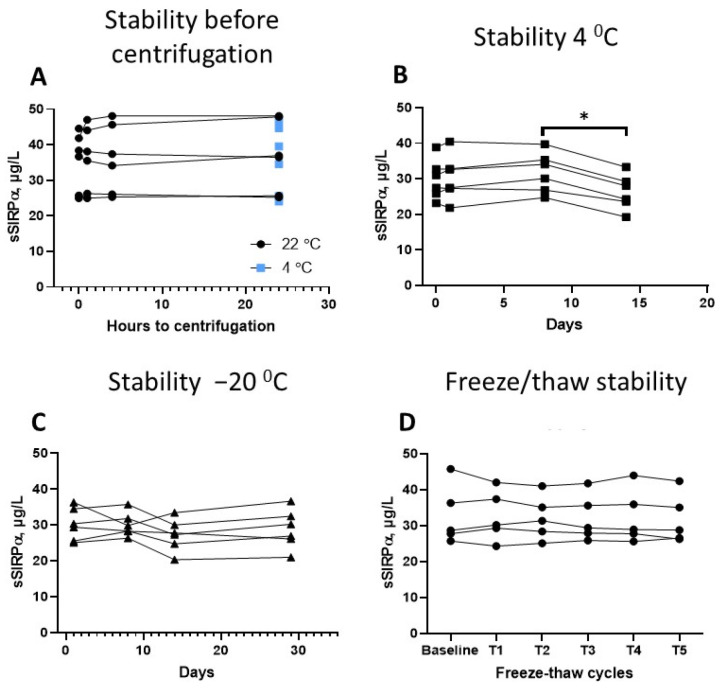
(**A**) Whole blood samples stored at room temperature or 4 °C were stable for up to 24 h before centrifugation. (**B**) Pipetted serum samples were stable at 4 °C for up to 8 days and underwent a small reduction in concentration between days 8 and 14 (*p* = 0.0313), * *p* < 0.05. (**C**) Pipetted serum samples were stable at −20 °C for up to 29 days (*p* = 0.3125). (**D**) Concentration of sSIRPα in pipetted serum was unaffected for at least five freeze–thaw cycles at −80 °C (*p* = 0.5347).

There was no difference between serum- and plasma-samples (*p* = 0.95). In pools of serum, sSIRPα was stable for more than 24 h at RT and 4 °C before centrifugation of blood-samples (Figure 4a). In pipetted serum, sSIRPα was stable for at least one week at 4 °C (Figure 4b), and for at least 29 days at −20 °C (Figure 4c). The concentration of sSIRPα was found to be stable through at least five rounds of freezing and thawing (Figure 4d).

### 3.5. Serum Concentration of sSIRPα in Healthy Individuals

Soluble SIRPα was measured in serum from 120 healthy blood donors (60 men, 60 women, age 18–69) and was clearly detectable in all samples. The samples had levels between 17.4 and 54.6 µg/L (median 28.7) and showed a log-Gaussian distribution. A parametric 95% reference interval was established from log-data to 19.8 µg/L (90% CI 18.8–20.8) to 41.1 µg/L (90% CI 39.2–43.1). There was no positive correlation between sSIRPα and age (R^2^ = 0.01, *p* = 0.22). Men had marginally higher levels than women (mean 29.5 vs. 28.5 µg/L, *p* = 0.017).

## 4. Discussion

In this work, we show that SIRPα is present as a stable soluble protein in human blood which we denote sSIRPα. A soluble form of SIRPα has previously been detected in vitro [11,13,14], but to our knowledge, this is the first time sSIRPα has been detected in the human circulation.

It is possible that this soluble form of SIRPα may function as a biomarker of the activation of TAMs during immunotherapy targeting the innate immune system, similar to the use of sPD1 during T-cell targeted therapy [19].

SIRPα is expressed on neurons and myelomonocytic cells [8,9]. Especially its role in anti-inflammatory M2-like TAMs is of interest. Here, it transmits a “don’t eat me signal” upon binding to the phagocytosis checkpoint CD47 on tumor cells. In accordance with this we observed a significantly lower SIRPα expression on inflammatory M1 MDMs in parallel with an increase in soluble SIRPα in the adjacent cell medium. This is, at least partly, due to an inflammatory activation of sheddases, of which ADAM10 seems to be involved in SIRPα shedding in humans [12]. The remaining intracellular fragment is cleaved further by γ-secretase. The accumulated intracellular product triggers phosphorylation of IKKα/β, which in turn activates NFκB and induces inflammation [14].

Soluble SIRPα was detectable in all investigated serum samples, and the concentration in serum increased after in vivo LPS challenge in healthy individuals. Although the increase was significant, it was less pronounced and acute than was seen for the endocytic macrophage receptor CD163. This may be due to a more direct LPS activation of the ADAM17 enzyme responsible for sCD163 shedding [20]. Nevertheless, the sSIRPα increase in response to LPS indicates that macrophage activation is reflected in circulating levels of sSIRPα.

As TAMs expressing SIRPα are abundant in cancer, sSIRPα may function as a biomarker, and we envision two potential uses of sSIRPα. Firstly, high pre-treatment levels of sSIRPα could potentially signal a poor prognosis reflecting sSIRPα constitutively released by high numbers of TAMs. This is supported by SIRPα being constitutively released by MDMs and by recent evidence linking high SIRPα expression to poor survival in several tumors [11,21,22,23,24]. Secondly, a rise in sSIRPα levels after initiation of immunotherapy may reflect the level of immune activation achieved, and thus predict the successfulness of the treatment. This is supported by the observed increase in sSIRPα after pro-inflammatory activation both in vitro and in vivo. Importantly, the activation may also shift the balance between soluble and cell-bound SIRPα, resulting in a decreased CD47 binding of cellular SIRPα due to blockage by increasing sSIRPα [25,26].

A limitation of our study is that the precise identity of sSIRPα in serum is not known. In Western blotting, the size of the immunologically measured protein corresponds to the extracellular part of SIRPα. This could be strengthened by a comparison to sSIRPα from macrophage cell cultures and characterization of purified serum sSIRPα by mass-spectrometry [18].

Our results pave the way for future studies exploring the clinical use of sSIRPα. Overall sSIRPα proved to be a very stable protein in the blood, facilitating blood-sampling and biobanking. Additionally, to help transferability of results across assays, we established a reference interval for the healthy population, using blood donor samples traceable to an international reference serum.

In conclusion, we have demonstrated that soluble SIRPα is present in human serum (denoted sSIRPα); it is stable, and it can be measured using the described validated assay. It is produced constitutively by MDMs, and the concentration of sSIRPα increases upon macrophage activation both in vitro and in vivo. sSIRPα may therefore function as a biomarker for the activity of TAMs and for monitoring immunotherapy, which is now being investigated in several clinical studies.

## Figures and Tables

**Table 1:** SIRPα flowcytometry.

MDM Subtype	Mean SIRPα (MFI)	MFI Range	Test (*p*-Value)	Relative Difference (%)
M0	22,982	(15,835; 32,596)	0.078	45.83
M1	15,760	(8,010; 20,130)	-	-
M2a	28,522	(16,921; 39,051)	0.065	80.97
M2c	20,631	(16,434; 26,940)	0.243	30.90

Mean Fluorescent Intensity (MFI) range is presented as (lowest value; highest value). Repeated Measures one way ANOVA followed by Tukey’s multiple comparison test compared to M1 MDMs.

**Table 2:** sSIRPα ELISA.

MDM Subtype	Mean sSIRPα (µg/L)	sSRIPα Range	Test (*p*-Value)	Relative Difference (%)
M0	5.43	(3.87; 7.37)	0.0024 **	−40.09
M1	9.07	(8.13; 10.5)	-	-
M2a	6.54	(4.26; 8.47)	0.0229 *	−27.92
M2c	6.07	(4.48;7.90)	0.0494 *	−33.12

sSIRPα range is presented as (lowest value;highest value). Repeated Measures one way ANOVA followed by Tukey’s multiple comparison test compared to M1 MDMs. * *p* < 0.05; ** *p* < 0.01.

## Data Availability

The data presented in this study are available on request from the corresponding author.

## Supplementary Materials

The following supporting information can be downloaded at: https://www.mdpi.com/article/10.3390/biom12070937/s1, Figure S1: Gating strategy; Figure S2: Polarization of MDM.
