# Supplementary material for: A New Serum Macrophage Checkpoint Biomarker for Innate Immunotherapy: Soluble Signal-Regulatory Protein Alpha (sSIRPα)
Source: Biomolecules. 2022 Jul 4;12(7):937. doi: 10.3390/biom12070937 (PMC9312483; doi:10.3390/biom12070937)
Supplement: Supplementary file 1 [file biomolecules-12-00937-s001.zip › Figure S1.pdf]

Supplemental figure 1

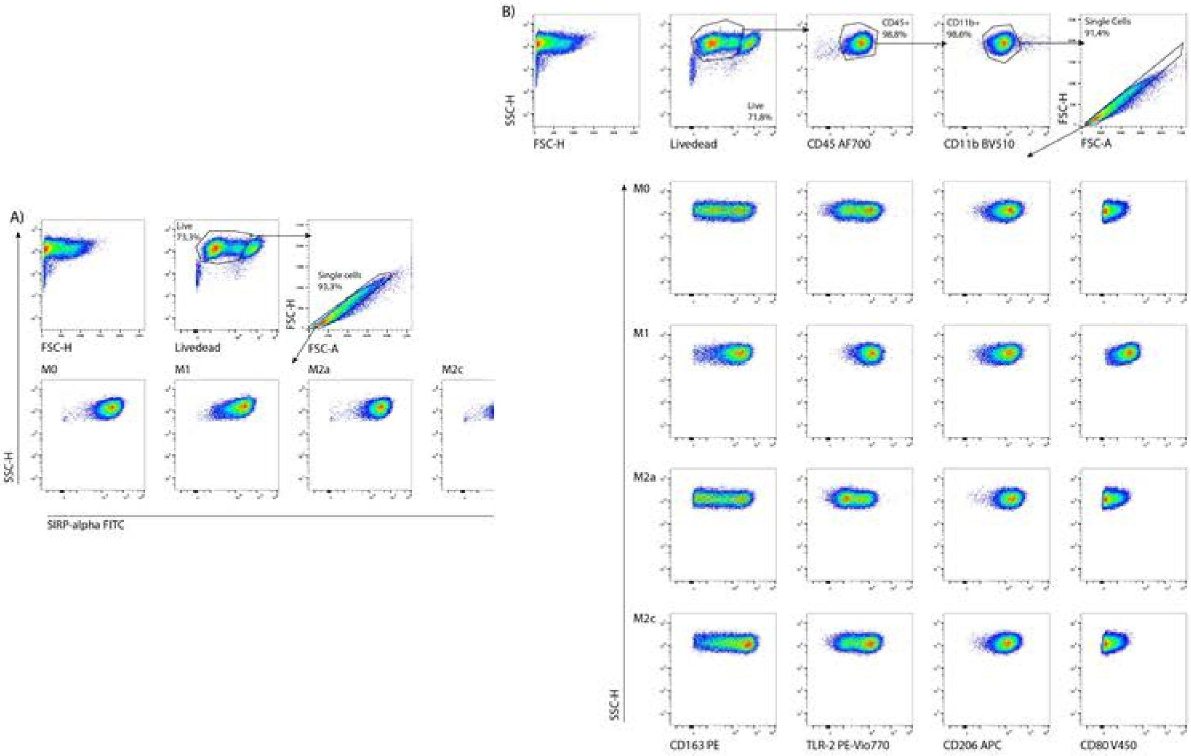

Supplemental Figure S1. **Gating strategy. A)** Cells to assess SIRP-alpha expression were gated as live/single cells. Representative plots for SIRP-alpha expression for each polarization. **B)** Cells to verify MDM polarization were gated as live/CD45+/CD11b+/single cells. Representative plots for CD163, TLR2, CD206 and CD80 for each polarization.
