# Supplementary material for: A New Serum Macrophage Checkpoint Biomarker for Innate Immunotherapy: Soluble Signal-Regulatory Protein Alpha (sSIRPα)
Source: Biomolecules. 2022 Jul 4;12(7):937. doi: 10.3390/biom12070937 (PMC9312483; doi:10.3390/biom12070937)
Supplement: Supplementary file 1 [file biomolecules-12-00937-s001.zip › Figure S2.pdf]

Supplemental figure 2

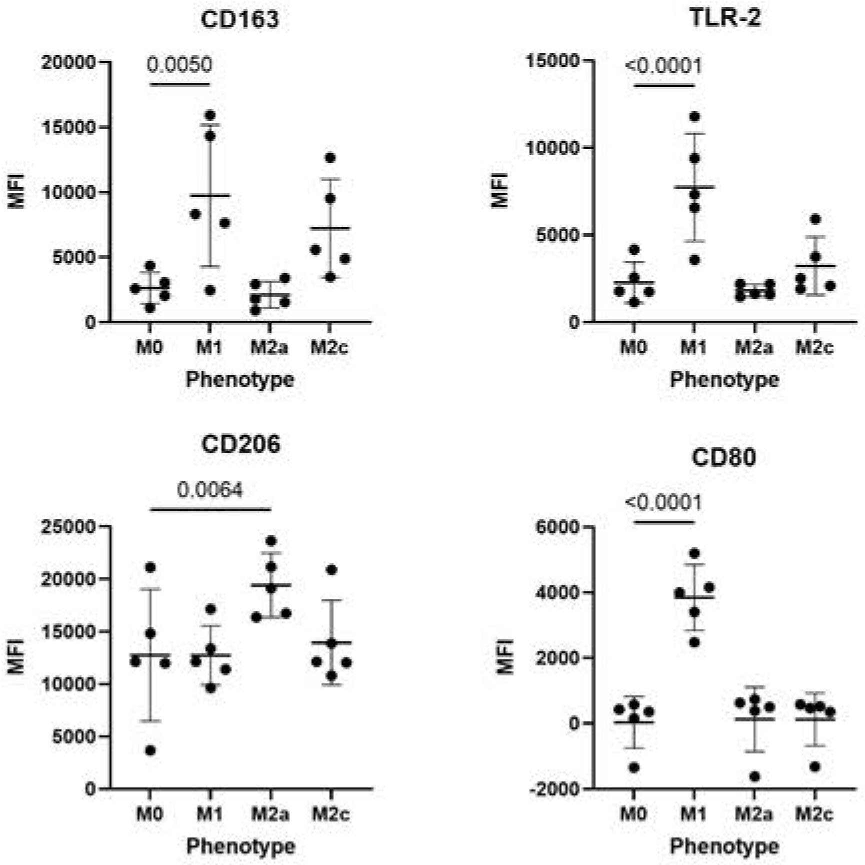

Supplemental Figrue S2. Polarization of MDM. Monocyte derived macrophages (M0) were polarized into M1(LPS, IFN-γ), M2a (IL-4, IL-13) and M2c (IL-10) macrophage subtypes. To validate the MDM polarization, the expression of cell surface markers CD163, TLR-2, CD206 and CD80 were investigated for each subtype using flowcytometry. Repeated measures one way ANOVA with Dunnet's multiple comparisons test.
